# The Production of Matchout-Deuterated Cholesterol and the Study of Bilayer-Cholesterol Interactions

**DOI:** 10.1038/s41598-019-41439-z

**Published:** 2019-03-26

**Authors:** Sarah Waldie, Martine Moulin, Lionel Porcar, Harald Pichler, Gernot A. Strohmeier, Maximilian Skoda, V. Trevor Forsyth, Michael Haertlein, Selma Maric, Marité Cárdenas

**Affiliations:** 1 Institut Laue-Langevin, 71 Avenue des Martyrs, 38042 Grenoble, Cedex 9 France; 2 Biofilm-Research Centre for Biointerfaces and Biomedical Science Department, Faculty of Health and Society, Malmo University, Malmo, 20506 Sweden; 3 Austrian Centre of Industrial Biotechnology, Petersgasse 14, 8010 Graz, Austria; 4 Graz University of Technology, Institute of Molecular Biotechnology, NAWI Graz, BioTechMed Graz, Petersgasse 14, 8010 Graz, Austria; 5 Graz University of Technology, Institute of Organic Chemistry, NAWI Graz, Stremayrgasse 9, 8010 Graz, Austria; 6 Rutherford Appleton Laboratory, Harwell, Didcot OX11 0QX UK; 7 Life Sciences Department, Faculty of Natural Sciences, Keele University, Staffordshire, ST5 5BG UK; 8 MAX IV Laboratory, Fotongatan 2, 225 92 Lund, Sweden

## Abstract

The deuteration of biomolecules provides advanced opportunities for neutron scattering studies. For low resolution studies using techniques such as small-angle neutron scattering and neutron reflection, the level of deuteration of a sample can be varied to match the scattering length density of a specific D_2_O/H_2_O solvent mixture. This can be of major value in structural studies where specific regions of a complex system can be highlighted, and others rendered invisible. This is especially useful in analyses of the structure and dynamics of membrane components. In mammalian membranes, the presence of cholesterol is crucial in modulating the properties of lipids and in their interaction with proteins. Here, a protocol is described for the production of partially deuterated cholesterol which has a neutron scattering length density that matches that of 100% D_2_O solvent (hereby named matchout cholesterol). The level of deuteration was determined by mass spectrometry and nuclear magnetic resonance. The cholesterol match-point was verified experimentally using small angle neutron scattering. The matchout cholesterol was used to investigate the incorporation of cholesterol in various phosphatidylcholine supported lipid bilayers by neutron reflectometry. The study included both saturated and unsaturated lipids, as well as lipids with varying chain lengths. It was found that cholesterol is distributed asymmetrically within the bilayer, positioned closer to the headgroups of the lipids than to the middle of the tail core, regardless of the phosphatidylcholine species.

## Introduction

Cholesterol is a key component of cellular membranes that modulates the structure and organisation of the lipid bilayer including the formation of so-called lipid rafts^1^. Lipid rafts are naturally existing nano-sized domains which are thought to regulate transmembrane proteins, some of which are linked to signalling mechanisms throughout the membrane^2,3^. Cholesterol provides rigidity in membranes by inducing liquid-ordered phases, and also affects structure by bringing about changes in the thickness of lipid monolayers (by condensation)^4,5^ and bilayers (by altering the fluid and gel phases)^6–8^.

It has recently been suggested that cholesterol positions itself in a lipid bilayer in a manner that depends on its environment: in polyunsaturated or very thin saturated bilayers, cholesterol will preferentially locate towards the core of the bilayer^9–11^, while for thicker bilayers made of saturated or monounsaturated lipids, the cholesterol molecules will sit closer to the hydrophilic head groups of the lipid core^11^. It was suggested that the displacement of cholesterol towards the core is due to a solubility limit related to the order parameter of the lipids in which it is found^9,10,12^. This is quite a surprising result that requires further investigation. An excellent technique to study the structure and composition of thin membranes is neutron reflectometry (NR), as it is a highly sensitive structural tool with a resolution of a few angstroms perpendicular to the plane of the solid-liquid interface^13,14^.

The availability of deuterated lipids is crucial for neutron scattering studies that exploit the very large difference in the scattering length densities (*ρ*) of protium and deuterium. This approach provides a way to study the positioning of a lipid molecule with respect to different environments and has successfully been used for the study of several systems including mixtures of deuterated phospholipids^15–17^ and unlabelled sterols^18^. The deployment of D_2_O matched-deuteration, referred to herein as simply matchout-deuteration, (in contrast to *per*deuteration) in neutron structural studies has been discussed recently by Dunne *et al*.^19^ and Haertlein *et al*.^20^. This approach is based on the concept of deuterating specific molecules (in this case cholesterol) to a level that endows them with a *ρ* that is equal to that of pure D_2_O, and as such is invisible to small angle neutron scattering (SANS) or NR at this solvent condition. The matchout-deuteration regime allows more sophisticated neutron-based experiments to be designed whereby within a single sample preparation, different components of a complex can be rendered “invisible” to neutron scattering techniques such as SANS and NR. This matchout deuteration technique allows components to be invisible to a *per*deuterated environment, however different mixtures of H_2_O and D_2_O can give the same effect when the *ρ* of the solvent mixture matches the *ρ* of a component of a system (typically having a value in between that of H_2_O and D_2_O) which is in turn rendered invisible. The benefits of this contrast matching allows a simplification of a complex system by focussing on certain parts and not having any extra scattering from those parts that are not of interest. Using solely a mixture of H_2_O and D_2_O to contrast match components might not be sufficient, for example in the case that the *ρ* values for the phospholipids compared to non-deuterated cholesterol are very similar. *Per*deuteration gives a different advantage, in that it provides maximum contrast to a non-deuterated solvent background. This is beneficial if a greater signal is necessary or if a single component sample is under investigation.

Despite the benefits of molecular deuteration in scattering techniques, there are some considerations to bear in mind; namely if the deuteration of lipid components truly reflects the same system when non-deuterated. Deuteration is known to affect the phase transition temperature of phospholipids^21,22^ and the high resolution structural composition of lipid systems^22,23^. For low resolution studies, however, hydrogenous and deuterated lipid systems are often considered to be interchangeable.

In this work, a new protocol based on *in vivo* biosynthetic methods for the production of deuterated cholesterol is presented. The cholesterol was characterised by mass spectrometry (MS), nuclear magnetic resonance (NMR), and SANS. Moreover, the matchout cholesterol is used to further investigate its location in different lipid bilayers by NR. Specifically, we use supported lipid bilayers (SLBs) of matchout deuterated cholesterol-containing phosphatidylcholine (PC) with various saturated lengths (C14:0 DMPC and C12:0 DLPC) and level of unsaturation (1-palmitoyl-2-oleoyl-glycero-3-phosphocholine, C16:0,18:1 or POPC).

## Results

### Growth and Fermentation Conditions for the Production of Matchout-Deuterated Cholesterol

*Per*deuteration of cholesterol can be achieved in high cell density cultures using lipo engineered strains of *Pichia pastoris* grown in a medium containing the solvent (D_2_O) and a carbon source (d8-glycerol) in their deuterated forms^24^. To reduce the deuteration level to a level required for matchout labelling, a medium containing D_2_O and unlabelled glycerol was used. Cells were adapted to growth in the deuterated medium prior to high cell density cultures. At the end of the fermenter run, the final optical density measured at 600 nm (OD_600_) was 42 and the cellular wet weight (CWW) was 37 g, giving a final yield of 66.5 mg of matchout-deuterated cholesterol for 1 litre of culture.

### Structural Characterisation of Matchout-Deuterated Cholesterol

**Figure 1:**
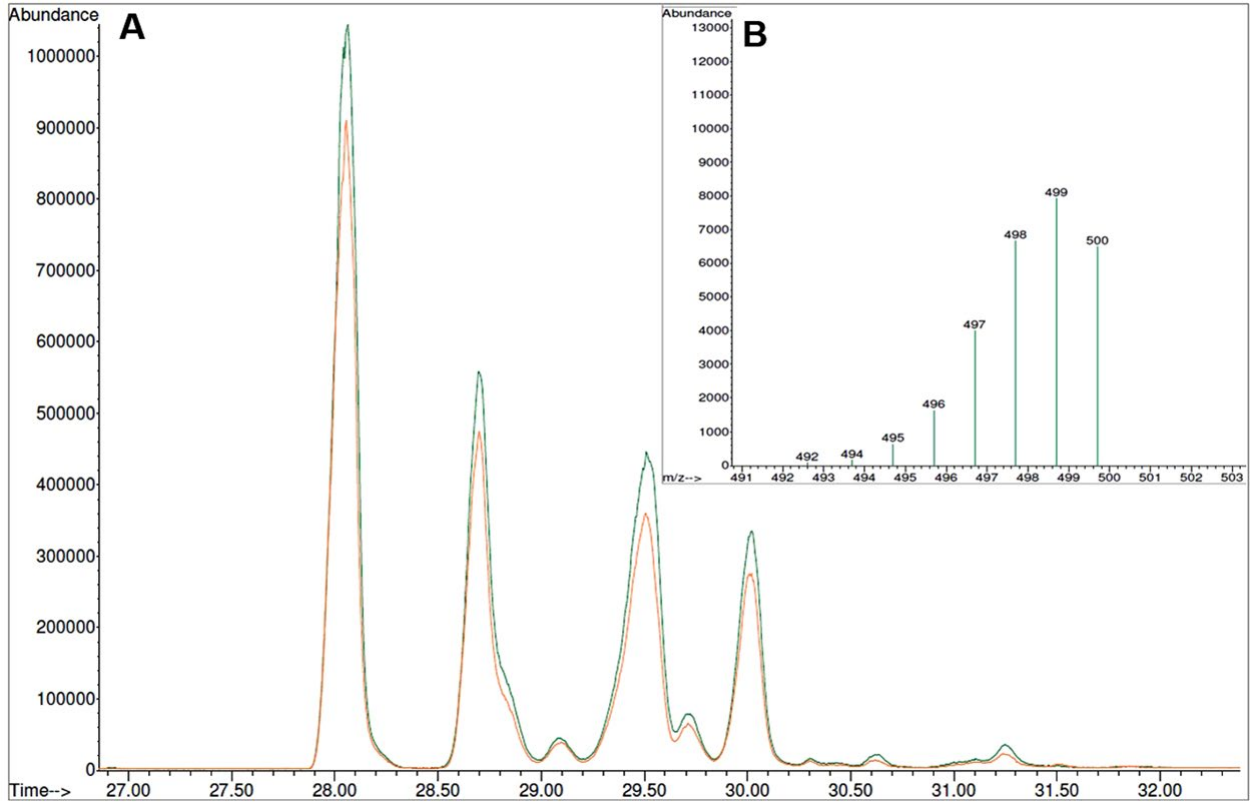
GC-MS analysis of the sterol components extracted from the partially deuterated cell paste. (**A**) GC-MS chromatogram showing the total sterol components, each sample was run in duplicate (green and orange curves). The first and main peak corresponds to cholesterol. Further details relating to the remaining peaks are given in Table 1. (**B**) MS spectrum for the isolated cholesterol component showing the main mass peak.

The analysis of the lipid fractions from the purified cell paste, as carried out by gas chromatography coupled with mass spectrometry (GC-MS) (in duplicate – green and orange curves), showed a peak eluting at 28.1 min (Fig. 1A). This peak corresponds to cholesterol. The retention times and composition as estimated by the area under the chromatographic peaks are given in Table 1. The presence of cholesterol is demonstrated by the MS spectrum that shows a main peak at 499 m/z (Fig. 1B). This main peak corresponds to cholesterol with a mass of 427 g/mol. The cholesterol shown has undergone silylation during the derivatisation process, where a trimethylsilyl moiety with a mass of 72 g/mol leading to the total mass of the complex at 499 m/z was added. This step is required to ensure sufficient volatility of the sample, enabling accurate and efficient GC-MS analysis^25^. The remaining peaks were assigned by comparison to a non-deuterated sample, as was reported previously^24^. These sterols also had additional peaks in their MS chromatograms arising from various intermediates during their synthesis.

**Table 1:** Table of sterol components in GC-MS sample.

Peak	Compound	Retention Time (RT) (min)	Rel. RT	% of Total Sterols
1	Cholesterol	28.1	1.000	47.1 ± 1.3
2	7-DHC	28.7	1.023	23.2 ± 0.3
3	Zymosterol	29.1	1.037	1.3 ± 0.0
4	Cholesta-5,7,14,24(25)-tetraenol	29.5	1.052	26.1 ± 1.4
5	Cholesta-7,24(25)-dienol	29.7	1.059	2.2 ± 0.2
6	Ergosterol (IS)	30.0	1.070	
Total sterols	4.5 ± 0.2 ug/mg (CWW)			

**Figure 2:**
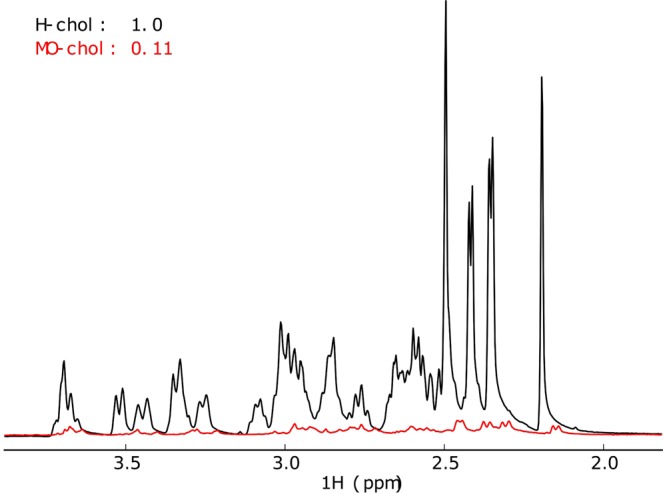
^1^H-NMR spectra for non-deuterated and matchout-deuterated cholesterol in black and red respectively. These spectra were measured in CD_3_OD and at a concentration of 2 mM. The spectra were collected on a Bruker 1D NMR spectrometer at 600 MHz.

The cholesterol produced, labelled MO-chol in Fig. 2 and Table 2, was found to have an 89% level of deuteration by ^1^H-NMR. This level of deuteration accounts for 41 deuterium atoms and 5 protiums, giving a mass of 427 g/mol; this is supported by the GC-MS data.

**Table 2:** Summary of the experimentally determined CMPs and *ρ* values obtained for the lipid vesicles.

POPC	% POPC	% CHOL	*ρ*_*POPC*_ × 10^−6^ Å^−2^	*ρ*_*MO-CHOL*_ × 10^−6^ Å^−2^	*CMP*_*TOT*_	*CMP*_*MO-CHOL*_
Without MO-CHOL	100	0	0.44 ± 0.02		15 ± 1	
With MO-CHOL	60	40	0.44 ± 0.02	6.5 ± 0.2	37 ± 2	101 ± 2

### Contrast Match Point Determination

**Figure 3:**
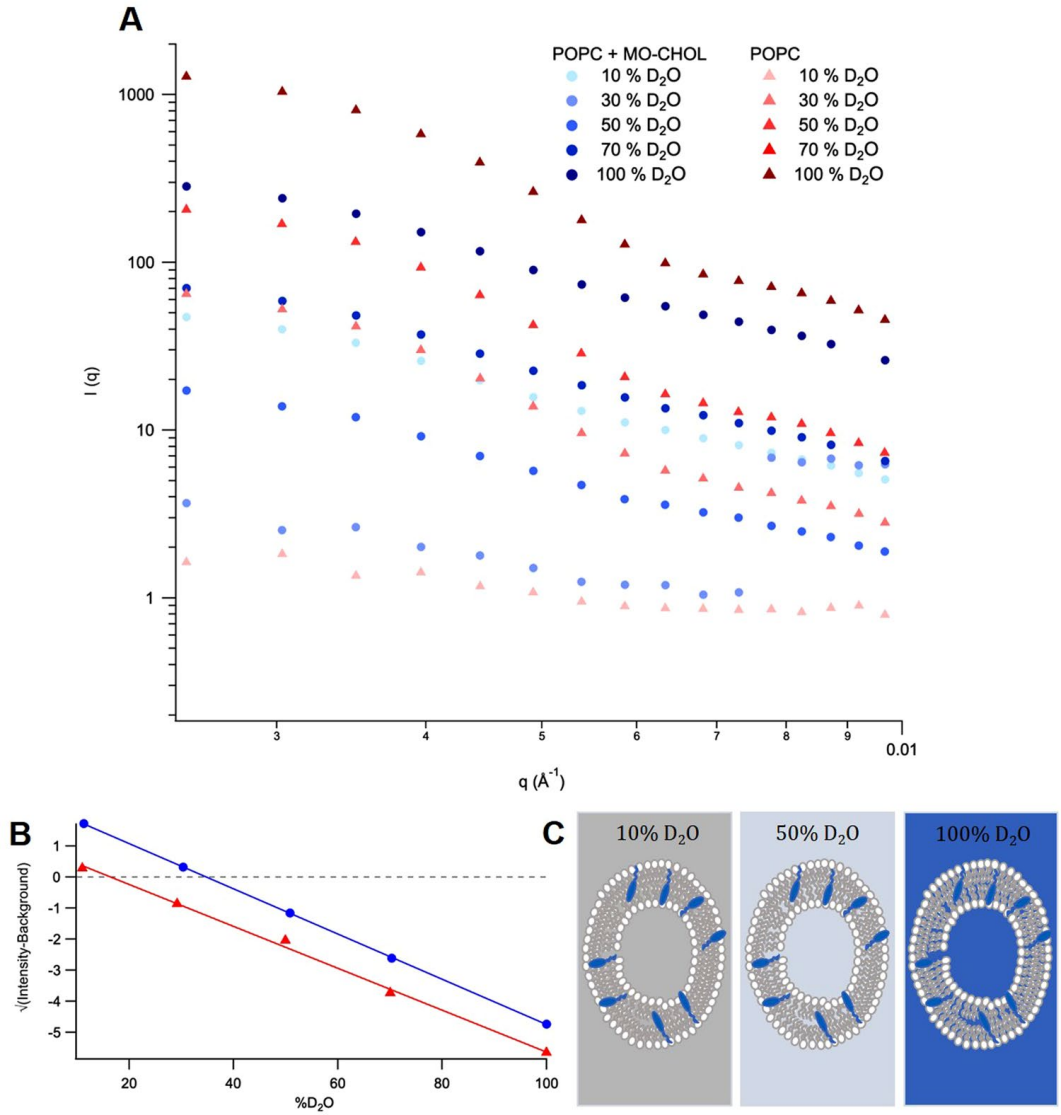
(**A**) The SANS spectra for the non-deuterated POPC series in the absence (red triangles) and presence (blue circles) of matchout cholesterol (MO-CHOL) in 10%, 30%, 50%, 70% and 100% D_2_O (v/v) contrasts. (**B**) The contrast match point series measured for non-deuterated POPC in the absence (red triangles) and presence (blue circles) of matchout cholesterol, measured in 10%, 30%, 50%, 70% and 100% (v/v) D_2_O contrasts. The lines are a linear fit to the data given by the symbols. (**C**) Pictorial representation of the model for the vesicles used in the SANS matchout series with POPC in the presence of 40 mol% cholesterol at contrasts of 10% D_2_O matching out the lipids tails, 50% D_2_O showing all components of the vesicles and 100% D_2_O matching out the cholesterol.

The scattering intensities of each series (either pure non-deuterated POPC or its mixtures with matchout cholesterol) are shown in Fig. 3A. The contrast match point (CMP) was determined for the composition of the solvent at y = 0 in Fig. 3B. The CMP for non-deuterated POPC including 40 mol% matchout cholesterol is an overall value and corresponds to the presence of both lipids and cholesterol.

Using the CMP for both series and Eqs 3–5 the CMP for cholesterol alone was found to be 101%, corresponding to a *ρ* value of 6.5 × 10^−6^ Å^−2^, as summarised in Table 2. The value obtained for the *ρ* is in excellent agreement with both the MS and NMR data. The errors given were determined using standard deviation analysis.

### Structure and Localisation of Matchout-Deuterated Cholesterol in Saturated and Monounsaturated Phospholipid Bilayers

**Figure 4:**
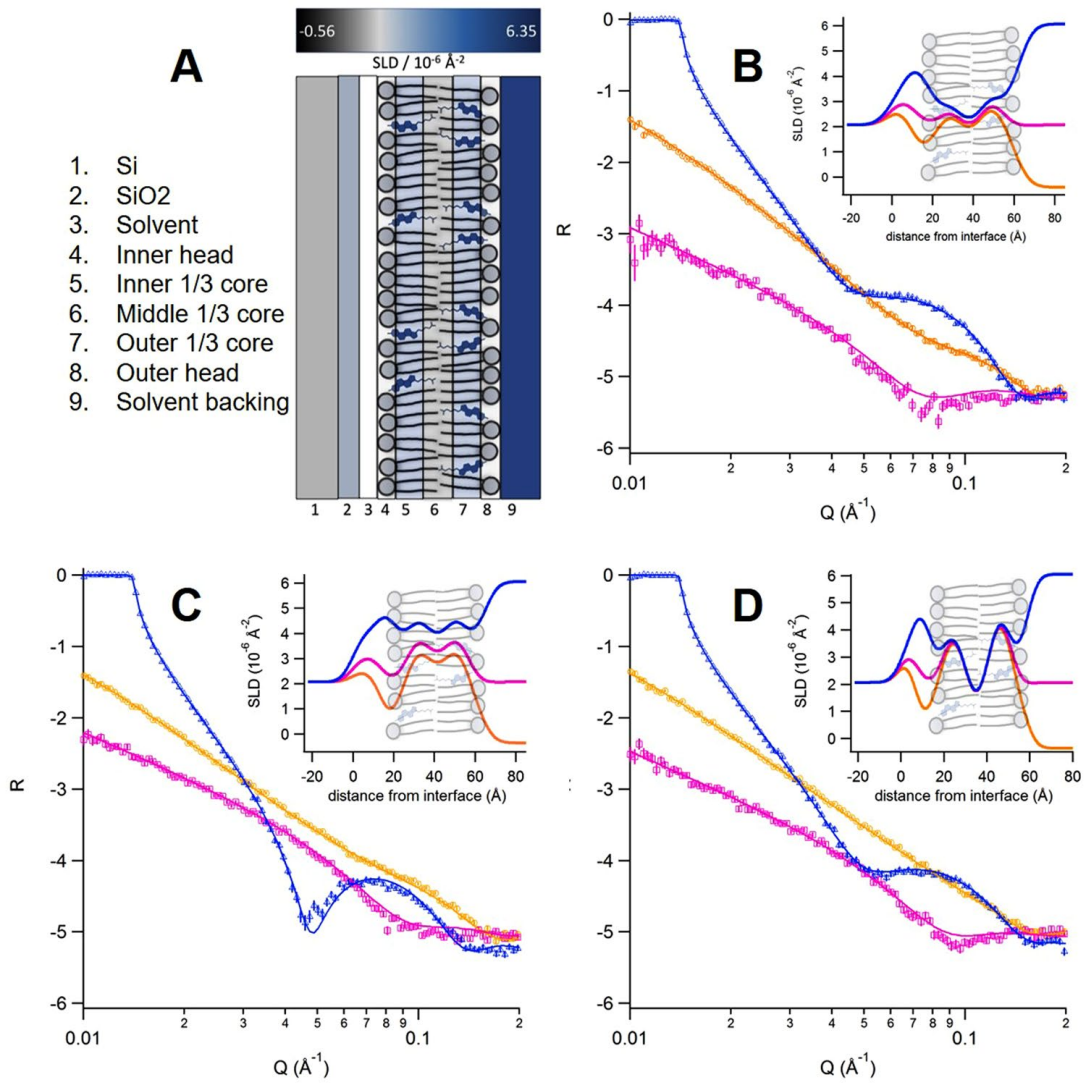
(**A**) Five layer model used to fit the SLBs at the SiO_2_-aqueous interface, and reflectivity curves for cholesterol-containing (**B**) DMPC, (**C**) DLPC and (**D**) POPC SLBs. The SLBs were formed by vesicle fusion at 37 °C using a vesicle composition of 60 mol% PC and 40 mol% cholesterol. The blue triangle, orange circle and pink square curves coincide with different contrasts (100% deuterated Tris buffer, 100% non-deuterated Tris buffer and cmSi respectively). The insets in (**B**–**D**) show the *ρ* profile for the best fits to the five layer model.

Three different SLBs were studied using NR to determine the positioning of the cholesterol within the bilayer: non-deuterated DMPC, POPC and DLPC each containing 40 mol% matchout-deuterated cholesterol. The three Tris buffer contrasts in D_2_O, H_2_O and a mixture giving the same *ρ* to contrast match the silicon block (cmSi) gave structural and compositional information on the bilayer and the data were fitted to a model as shown in Fig. 4A. The model includes the silicon block with a small silicon dioxide layer in contact with a small solvent layer followed by the bilayer. The bilayer is broken up into five-layers: an inner head group, three distinct tail regions and an outer head group exposed to the solvent contrast. The three sections of the tail core allow for an asymmetric distribution of cholesterol, as reported previously^26^. The thickness, coverage and roughness of the tail regions were all constrained during the fitting process to be symmetric (each of these parameters were kept equal throughout all three core layers) to limit the number of variables. However, the *ρ* for each section of the core was allowed to vary, granting the distribution of cholesterol throughout the bilayer.

Alternative models with only one or two layers in the lipid core were also tried. However, the fits were not satisfactory, suggesting an oversimplification of the composition of the SLB, assumed to be symmetrical and uniform throughout the core. A five-layer model was therefore necessary to understand where the cholesterol was located in the core - whether it accumulates in the centre of the bilayer core or positions itself at the edges of the bilayer close to the headgroups as seen previously^11,26^.

Figure 4B–D shows the NR curves for the three different SLBs studied, in each of the three isotopic contrasts (D_2_O, H_2_O and cmSi, in blue, pink and orange respectively). The best fitting parameters are given in Table 3. The total (and core) thickness for the bilayers are 45 (29.7) Å, 46 (31.5) Å and 44 (30.3) Å for the cholesterol-containing DMPC, POPC and DLPC SLBs respectively. In all three SLBs, the outer regions of the lipid core are enriched with cholesterol; this can be seen with the increase in *ρ* in the outer regions of the core, corresponding to the increased *ρ* value of the cholesterol compared to the lipid tails alone. In the DMPC bilayer, the inner (that closer to the Si block surface) and outer sections of the lipid core present an enrichment in cholesterol with 52 mol% and 61 mol% while only 20 mol% cholesterol is present in the central region of the lipid core. This gives an average of 45 mol% cholesterol content in the SLB, which is expected and very close to the nominal value of the parent vesicles used for the SLB formation. In the POPC bilayer, the inner and outer regions of the tail core contain 64 mol% and 75 mol% cholesterol while only 21 mol% is present in the middle section of the core, thus giving an overall 53 mol% cholesterol in the SLB. This is slightly higher than the nominal value to be expected from the parent vesicles. The DLPC SLB also contained enriched outer regions of the tail core with 72 mol% and 75 mol% for the inner and outer leaflets respectively, and only 47 mol% in the middle region. This in turn gives an average of 65 mol% cholesterol present in the DLPC SLB, which is again higher than the expected nominal value. Compositional deviations in SLBs from their parental vesicles for a two component system containing PC lipids close to its phase separation boundary and when using the vesicle fusion approach have been reported previously^15^.

**Table 3:** Structural parameters obtained from fitting of derived model to the reflectivity curves for the bilayers.

Sample		*ρ* × 10^−6^/Å^−2^	MO-Chol %	Thickness/Å	Coverage %	MMA/Å^2^
DMPC +40 mol% cholesterol	Inner Head	1.89*		8.2 ± 0.5**	92 ± 2**	43 ± 3
	Tail (3 sublayers)	3.2 ± 0.3	52 ± 5	29.7 ± 0.8**	93 ± 1**	50 ± 2
		1.0 ± 0.5	20 ± 9			54 ± 2
		3.8 ± 0.3**	61 ± 5			49 ± 2
	Outer Tail	1.89*		8.2 ± 0.5**	92 ± 2**	43 ± 3
			**Average:** **45** ± **11**	**Total:** **46** ± **1**		
POPC +40 mol% cholesterol	Inner Head	1.89*		7.2 ± 0.2**	87 ± 3**	52 ± 2
	Tail	4.06 ± 0.07	64 ± 1	31.5 ± 0.3**	99 ± 1**	47 ± 1
		1.11 ± 0.08	21 ± 1			56 ± 1
		4.83 ± 0.07**	75 ± 1			45 ± 1
	Outer Tail	1.89*		7.2 ± 0.2**	87 ± 3**	52 ± 2
			**Average:** **53** ± **2**	**Total:** **45.9** ± **0.4**		
DLPC +40 mol% cholesterol	Inner Head	1.89*		7.0 ± 0.3**	74 ± 3**	63 ± 4
	Tail	4.6 ± 0.2	72 ± 3	30.3 ± 0.5**	83 ± 1**	50 ± 2
		2.9 ± 0.2	47 ± 3			51 ± 2
		4.8 ± 0.1**	75 ± 2			50 ± 2
	Outer Tail	1.89*		7.0 ± 0.3**	74 ± 3**	63 ± 4
			**Average:** **65** ± **5**	**Total:** **44.4** ± **0.7**		

*Values kept constant during the fitting process.

**The errors are given in Table 3 and are calculated using a Monte-Carlo analysis as embedded in the motofit software^43^.

## Discussion

In this paper, a new protocol is presented for the production of matchout-deuterated cholesterol in a yeast host. The method is based on growing a genetically modified yeast strain^27^ in deuterated media using a non-deuterated carbon source (glycerol) to obtain the required level of deuteration. Both MS (Fig. 1) and ^1^H NMR (Fig. 2) suggest a level of deuteration of the cholesterol of 89%, corresponding to 41 deuterium and 5 protium atoms. The asymmetric distribution of ions for the main peak in the MS spectrum (Fig. 1B) implies no compounds have a higher deuteration level than 89% (there is otherwise a Gaussian distribution of deuteration around the main ion)^24,28^. This demonstrates the efficacy of the production method and its practical application for the matchout deuterium labelling that is often extremely helpful in neutron scattering work^29,30^. From the biosynthetic pathway, an outline of which can be seen in the Supplementary Information Scheme SI1, it is obvious that the presence of *per*deuterated solvent largely determines the level of deuteration in cholesterol. Since the non-deuterated glycerol is the only carbon source in the growth media, it provides the carbon backbone of cholesterol and all the original protiums. Certain protium atoms in this carbon backbone have a lower likelihood to undergo deuterium exchange during the biosynthesis process. The tautomerisation of Acetyl CoA (see Scheme 2 in Supplementary Information) results in an equilibrium of products which largely determines the level of deuteration in the final product, as it is during this step there is most uncertainty in the resulting hydrogen atoms. These equilibrium products react together to continue along the biosynthetic pathway. Therefore, from the biosynthesis of cholesterol we can expect that the use of non-deuterated glycerol as the sole carbon source will result in at least five protiums not being exchanged by deuterium - four present in methyl groups and one labile protium on the oxygen. The number of protium atoms incorporated into matchout cholesterol as determined by MS and NMR allowed the calculation of a theoretical *ρ* of 6.5 × 10^−6^ Å^−2^. The experimental SANS data (Fig. 3) confirmed the *ρ* of the matchout deuterated cholesterol preparation to be 6.5 × 10^−6^ Å^−2^. This corresponds to the (notional) CMP of 101% D_2_O solvent.

This D_2_O matchout cholesterol preparation was used to investigate the positioning of the cholesterol molecules within the leaflets of SLBs made of various PC lipids. NR was chosen due to it being a highly suitable technique for studying density profiles of lipid films in a direction perpendicular to an interface and having down to a few Angstroms resolution^13,14^. In a previous study, a five-layer model for SLBs made of natural, D_2_O matchout deuterated PC extracts has been used to show the asymmetrical distribution of both non-deuterated and *per*deuterated cholesterol throughout the lipid core and that the molecule is located close to the PC headgroups^26^. In that work, *per*deuterated cholesterol and non-deuterated cholesterol was mixed with D_2_O matched natural PC bilayers specifically to give contrast and highlight the cholesterol, whereas here we use non-deuterated bilayers and D_2_O matched cholesterol to ensure minimal signal from the cholesterol while being able to specifically compare the scattering from the lipid bilayers. An advantage of the previous work was the use of a lipid extract to determine the structure and positioning of cholesterol within a bilayer made of natural lipids. However, a drawback to the use of a natural lipid extract, and in turn an advantage of using singular lipid species in this instance, is the well-defined lipid volumes attainable and necessary for the detailed structural information extracted. A systematic study of single lipid structures is possible, in turn verifying the model previously determined. The 5 layer (head layer- 3 equally thick lipid core sublayers - head layer) model was used here to determine the location of cholesterol within the bilayer as a function of the length of saturated lipids (C12 vs C14) and the presence of unsaturation (POPC). This is relevant to recent NMR, MD and SANS analyses that suggest that the PC species determines the positioning of cholesterol within the lipid bilayer^11^. In particular, it has been suggested that the thickness of the bilayer core may determine the position and orientation of cholesterol - with thin bilayers forcing the cholesterol out of the bilayer structure and into the middle section of the lipid core: for cholesterol-containing lipid films where the half core thickness is shorter than the extended length of a cholesterol molecule (15 Å) the steroid moiety tilts and descends into the bilayer centre.

In the absence of (and presence of 10 mol%) cholesterol, the lipid bilayer core thickness was reported to be 30 Å^31^, 25.8 (34.1) Å^11,32^ and 21.8 (28.2) Å^11,32^ for POPC, DMPC and DLPC respectively at similar experimental conditions. Therefore, these SLBs should probe whether the bilayer core thickness affects the positioning of cholesterol within the lipid bilayer structure: the thicker DMPC and POPC bilayers should lead to cholesterol being closer to the headgroups, while DLPC should lead to cholesterol being gathered in the middle of the core.

The results obtained here show that the cholesterol was located nearer the headgroups in all of the SLBs studied, as can be seen from the difference in *ρ* values for the different regions in the hydrophobic core arising from the cholesterol-rich regions (insets in Fig. 4). The core thicknesses were found to be (within the experimental error) 30 Å for DMPC and DLPC, and slightly thicker (by 1.5 Å) for POPC. As already discussed, Marquardt *et al*.^11^ hypothesised a limit of 30 Å for the displacement of cholesterol from the upright positioning towards the centre of the bilayer core. Kučerka *et al*.^33^ found when polyunsaturated lipids alone in the presence of cholesterol induced the cholesterol positioning to be in the centre of the bilayer, whereas the co-addition of as few as 5 mol% DMPC induced the upright positioning of cholesterol near the lipid headgroups due to the increased level of order it provided. Both phenomena are in agreement with the data provided here whereby the high cholesterol content (>45 mol%) provides a high level of order in the bilayers thus inducing the cholesterol to remain in an upright position, regardless of saturation level or chain length.

The concentration of cholesterol present (65 mol%) in the DLPC SLB means the bilayer is likely to be in a liquid-ordered phase or even on the boundary of a crystalline and lamellar phase, which is cholesterol-rich, at 37 ^o^C^34^. This increase in cholesterol concentration could lead to the formation of crystalline cholesterol regions, especially near the headgroups. Similarly, the DMPC SLB contained 45 mol% cholesterol which would imply that the bilayer would be in a quite rigid liquid-ordered phase^35^.

For the cholesterol containing POPC bilayers, the sole unsaturated lipid studied, cholesterol was located at the head-tail interface. This is expected as it has been previously reported that for unsaturated bilayers the cholesterol tends to be located nearer the headgroups^26^. Interestingly, the phase of the cholesterol containing POPC bilayer was also likely to be liquid-ordered due to the slightly increased level of cholesterol. At 40 mol% cholesterol, the POPC-cholesterol phase diagram^36^ predicts the coexistence of the liquid-ordered and liquid-disordered phases at 37 ^o^C. However, the NR data showed slightly higher levels of cholesterol present (53 mol%) thus pushing the phase slightly over into the liquid-ordered region of the phase diagram. Earlier it has been found that the composition of SLB can differ from that of the nominal vesicles, and this has to do with differences in composition at the single vesicle level that are size dependent and is more marked for mixtures that are close to a phase transition boundary^15^.

The experiments performed by Marquardt *et al*.^11^ contained phospholipids with only 10 mol% cholesterol. Under these conditions, the bilayers are likely to be in the liquid-disordered phase. Hence, it would seem possible that the liquid crystalline phase of the cholesterol containing lipids may also play a key role in determining the positioning of the molecules in addition to the total thickness of the core or level of saturation of the core.

It is well known that *per*deuteration of lipids can impact their phase transition behaviour^22^. Recently, it was reported that the *per*deuteration of lipids reduced their bilayer thickness besides reducing their melting temperature. Even though the phase transition reported is not of that between a co-existing mixture of liquid disordered and liquid ordered to solely liquid ordered (as shown here), a similar effect is likely to be seen. However, it is important to note that it was also reported that the bilayer structure was not much altered away from the melting temperature. Their suggestion to overcome the differences induced by the deuteration is to use only partial deuteration to benefit from the difference in *ρ* without perturbing the bilayer itself significantly. The work here uses non-deuterated lipids and partially deuterated cholesterol which meets these requirements, thus implying there should be little interference and the use of the partially deuterated cholesterol is suitable to verify the previously established protocol and model.

Understanding the data presented here can help to determine the effect that cholesterol exerts on a bilayer, which can in turn give further insight into the function of cholesterol itself and how bilayers, and therefore cellular membranes, behave. This is particularly important for the structure and function of endothelial membranes (that contain about 20 mol% cholesterol^37^) and neural cells (that contain up to 40 mol% cholesterol^38,39^). Additional experiments using a wider range of cholesterol contents are needed to clarify the role of bilayer thickness for the distribution of cholesterol, however it should be emphasised that the accuracy of NR decreases sharply with reduced contrast (which would be the case of a SLB containing only 10 mol% cholesterol).

The availability of matchout-deuterated cholesterol opens up a range of additional possibilities of for membrane, cholesterol and protein structural studies. For example studies of matchout deuterated cholesterol may be used in combination with matchout deuterated PC^28^ to enable studies of the role that cholesterol plays in membrane protein structure and function in matchout deuterated nanodiscs^40^ and lipid-exchange studies in lipoproteins (unpublished results).

## Conclusions

A method has been developed for the production of matchout deuterated cholesterol for use in neutron scattering studies by the simple incorporation of non-deuterated glycerol in the otherwise deuterated growth medium, further demonstrating how the carbon source used in cultures can be used to control the level of deuteration in biomass.

The development of matchout-deuterated cholesterol allows for studies of its positioning within the lipid membrane, as demonstrated in the NR studies described here. It also opens up other options including the study of its regulation of membrane proteins^17^, its metabolism and uptake by cellular complexes such as lipoproteins, and other aspects that impact in terms of both structure and function in relation to other components in the cell membrane. In general, the inclusion of cholesterol in lipid bilayers gives a more realistic model membrane than phospholipids alone.

## Materials and Methods

### Theoretical D_2_O Match Point of Cholesterol

During the biosynthetic synthesis of cholesterol many protons are exchanged during enzymatic processes, therefore if a *per*deuterated growth medium is used the majority of protium atoms will be exchanged for deuterium atoms present in the medium. A *ρ* calculator was used to determine a theoretical *ρ* value for matchout cholesterol whereby only 5 hydrogen atoms would be required to remain as protium whereas the remaining 41 hydrogens could be exchanged to deuterium. Throughout the process most hydrogen atoms are exchanged meaning using a non-deuterated carbon source provides the correct level of non-deuteration in the overall solution mixture.

### Production of Yeast Biomass

Matchout-deuterated cholesterol was produced in the Deuteration Laboratory of ILL’s Life Sciences Group and purified using a protocol modified from that described by Moulin *et al*.^24^ for the production of perdeuterated cholesterol. In order to achieve a matchout level of deuteration, the previously genetically modified *Pichia pastoris* yeast strain was grown in the presence of non-deuterated glycerol as the sole carbon source in D_2_O minimal medium. One litre of deuterated basal salts medium (BSM) containing 10 g non-deuterated glycerol was inoculated (OD_600_ = 2.7) and grown in a three litre fermenter (Labfors, Infors) for a duration of three weeks. The pD level was monitored and adjusted by the addition of NaOD. After about a week when all of the glycerol had been consumed, the fed-batch phase was initiated by continuous feeding of a further 30 g of glycerol over 12 days.

### Purification of Partially Deuterated Cholesterol

Cholesterol was extracted and purified from the cell paste and analysed as reported previously^24^. The cell paste (batches between 27.5 and 34.5 g) was transferred into a 500 mL round-bottomed flask into which 60 g potassium hydroxide, 200 mL water, 100 mL methanol and 400 mg pyrogallol was added. The mixture was heated under gentle reflux for 3 h under a nitrogen atmosphere with minimal stirring to avoid foaming. After cooling to room temperature, 100 mL cyclohexane was added and the mixture stirred for another hour. Insoluble materials were then removed by filtration, once the mixture had cooled to room temperature, and the cyclohexane layer formed was separated using a separatory funnel. The methanolic solution was extracted a further two times each with 100 mL cyclohexane. The combined three cyclohexane extracts were washed with 100 mL water, dried over sodium sulphate and concentrated under reduced pressure. From a total of 123.7 g cell paste, 788 mg crude solid extract was obtained. To pre-purify the matchout-deuterated cholesterol, a flash chromatography on silica gel (silica gel 60, 0.040–0.063 mm, No. 109385, Marck, Darmstadt, Germany) using cyclohexane/ethyl acetate mixtures from 20:1 to 3:1 (v/v) was conducted. After removing the solvents under reduced pressure, 317 mg pre-purified material was obtained and, thus, the final HPLC separation could be done in a single run with the available equipment. Propan-1-ol proved to be a highly suitable solvent for dissolving the crude material in 2 mL volume. The cholesterol was isolated in pure form using a ThermoFisher UltiMate 3000 binary semi-preparative HPLC system equipped with a NUCLEODUR® 100-10 C18ec column (125 mm × 21 mm, 5 µm, Macherey-Nagel, Düren, Germany) attached to a VP 20/16 NUCLEODUR® C18ec guard column. Using an isocratic mixture consisting of acetonitrile/methanol (95:5) at a flow rate of 20 mL/min at 30 °C, the desired product eluted baseline-separated between 27.9 and 34.3 min as monitored by UV-detection at 210 nm. After removing the solvent under reduced pressure, 66.5 mg pure matchout-deuterated cholesterol was obtained. Analytical HPLC analysis of the final product purity was conducted on an Agilent 1100 system, equipped with a DAD detector and a NUCLEODUR® C18 gravity column (150 mm × 3 mm, 3 µm, No. 760083.30, Macherey-Nagel, Düren, Germany) using an isocratic mixture of acetonitrile/methanol 1:1 at a flow rate of 0.70 mL/min at 30 °C. The eluting peak at 8.24 min represents the desired product with a purity of >99% as checked by UV at 210 nm detection wavelength.

### Structural Characterisation of Partially Deuterated Cholesterol

#### Gas Chromatography Mass Spectrometry of Cholesterol

Gas chromatography coupled with mass spectrometry (GCMS) was carried out as reported previously^24^. 1 mL 0.2% pyrogallol in methanol and 400 μL 60% potassium hydroxide was used to resuspend 15 mg cell paste in Pyrex tubes. 10 μg ergosterol (1 mg/mL) were used as an internal standard and the sample was saponified at 90 °C for 2 h. The sample was extracted with *n*-heptane three times over and dried under a constant stream of nitrogen. 10 μL pyridine was used to dissolve the dried extracts, then derivatised with 10 μL *N*,*O*-bis(trimethylsilyl)trifluoracetamide. Samples were diluted in 50 μL ethyl acetate and analysed by GC-MS.

#### Nuclear Magnetic Resonance

The level of deuteration of the cholesterol was determined by ^1^H-NMR, measured at the Institut de Biologie Structurale (IBS), Grenoble, France. The samples were measured at a 4 mM concentration in deuterated chloroform. No internal standard was used. However, the non-deuterated cholesterol sample was set as a reference for 0% deuteration. The instrument used was a 600 MHz Bruker NMR spectrometer with an avance III HD console. A spectral width of 25 ppm was obtained, 32 scans were carried out with a relaxation delay of 20 sec and an acquisition time of 0.5 sec per scan. Bruker TopSpin software was used to analyse the data.

#### Lipid Vesicle and Supported Lipid Bilayer Formation

The lipids; 1-Palmitoyl-2-oleoyl-glycero-3-phosphocholine (POPC), 1,2-dimyristoyl-sn-glycero-3-phosphocholine (DMPC or C14 PC), 1,2-dilauroyl-sn-glycero-3-phosphocholine (DLPC or C12 PC), non-deuterated cholesterol and the extruder setup were purchased from Avanti Polar Lipids (Alabaster, AL).

For SANS, non-deuterated POPC lipid films were prepared in the presence and absence of 40 mol% partially deuterated cholesterol. The lipids were mixed from chloroform stocks of both POPC and cholesterol. The chloroform was evaporated using a nitrogen stream and the resulting films were put under vacuum overnight. The films were hydrated in D_2_O to a concentration of 50 mg/mL, extruded using a 100 nm filter and thereafter diluted 10-fold with varying H_2_O/D_2_O ratios. The lipids were used at a final concentration of 5 mg/mL in 10%, 30%, 50%, 70% and 100% D_2_O/H_2_O (v/v). By using a lipid stock prepared in 100% D_2_O for each lipid type, the same vesicle preparation was ensured for each series.

The lipids films required for NR were further hydrated in Milli-Q water to a concentration of 0.2 mg/mL and bath sonicated for 1 h. The lipid solutions were then tip sonicated for 5 min, at 10% power 5 sec on, 5 sec off, just before use. For optimum vesicle fusion, a 0.1 mg/mL solution of lipids in the presence of 2 mM CaCl_2_ was used^15^, by mixing equal volumes of the tip sonicated lipids with a 4 mM CaCl_2_ solution^26,41^. The sample was then introduced into the flow cell by a syringe injection and left to incubate at 37 °C for 20 min before rinsing the excess off in water followed by Tris buffer (50 mM Tris-HCl, 150 mM NaCl).

#### Neutron Scattering

SANS and NR data were collected where the scattered intensity as a function of the momentum transfer vector *q* = *4π* * *sin*(*θ*)/*λ*, were measured.

#### Determination of Contrast Match Point using SANS

SANS data were collected on the D22 instrument at the Institut Laue-Langevin (ILL) in Grenoble. The experiments were carried out at 25 °C using detector distances of 1.4, 5.6 and 17.6 m, providing a total *q* range of 0.002 < *q* < 0.56 Å^−1^. The CMP was obtained from the graph shown in Fig. 3B which plots √(intensity-background) against D_2_O concentration (*ϕ*). Here, the intensity value corresponds to the average of the intensities between 0.003 < q < 0.012 Å^−1^ and the background value corresponds to the average of the intensities between 0.45 < q < 0.55 Å^−1^. The data were corrected for the empty cell, background and used in absolute scale compared to the direct beam measurement. The cell thickness was 1 mm quartz glass Helma SANS cuvettes and the data reduction was carried out using GRASP.

The scattered intensity, *I*(*q*), of a vesicle solution is related to the form factor, *P*(*q*)_*lipid*_, volume, *V*_*lipid*_, number density, *n*, of the vesicles, and the difference in *ρ* between the vesicles (*ρ*_*lipid*_) and the solvent (*ρ*_*solvent*_):

1 $$I(q)=n\ast {V}_{lipid}^{2}\ast ({\rho }_{lipid}-{\rho }_{solvent})2\ast P{(q)}_{lipid}+{I}_{incoherent}$$

where *ρ* is given by the total sum of scattering lengths (*b*_*i*_) over the total volume (*V*):

2 $$(\rho )={\rm{\Sigma }}{b}_{i}/V$$

From the contrast match point (CMP), the *ρ* of sample can be acquired using the equation:

3 $${\rho }_{sample}=CM{P}_{TOT}\ast {\rho }_{D2O}+(1-CM{P}_{TOT})\ast {\rho }_{H2O}$$

For the two component systems, the overall *ρ* can be broken down into the following equation that combines each components *ρ* and volume ratio (*ϕ*):

4 $${\rho }_{sample}={\varphi }_{POPC}\ast {\rho }_{POPC}+{\varphi }_{CHOL}\ast {\rho }_{CHOL}$$

For the two component systems, the CMP can be broken down into the following equation that combines component CMP and *ϕ*:

5 $$CM{P}_{TOT}={\varphi }_{POPC}\ast CM{P}_{POPC}+{\varphi }_{CHOL}\ast CM{P}_{CHOL}$$

By rearranging this equation, the *ρ* of the cholesterol can be found from the overall CMP and the subsequent overall *ρ*.

#### Neutron Reflectometry Study

NR data were collected on the INTER instrument at the ISIS neutron facility, Didcot, UK^42^, and fitted using the MOTOFIT package^43^. The experiments were carried out at 37 °C using bespoke solid-liquid flow cells. The resolution was set to Δ*q*/*q* = 3% and the incident angles used were 0.7° and 2.3°. The area illuminated by the neutron beam was 30 × 60 mm^2^. The polyether ether ketone (PEEK) components of the cell were cleaned thoroughly using bath sonication in 2% (v/v) Hellmanex twice and MilliQ water, with rinsing in MilliQ water between each sonication. The Silicon (111) blocks used for depositing the bilayer were cleaned using Piranha solution (H_2_SO_4_/H_2_O_2_, 7:3) for 10 min at 80 °C before thorough rinsing with MilliQ water.

The DMPC, POPC and DLPC all containing 40 mol% matchout deuterated cholesterol SLBs were deposited in 2 mM CaCl_2_ and measured in three contrasts: D_2_O, H_2_O and a mixture which contrasts matches the silicon (cmSi) using Tris buffer, as previously mentioned.

## Supplementary information

Supplementary Information
