# Supplementary material for: The Production of Matchout-Deuterated Cholesterol and the Study of Bilayer-Cholesterol Interactions
Source: Sci Rep. 2019 Mar 26;9:5118. doi: 10.1038/s41598-019-41439-z (PMC6435723; doi:10.1038/s41598-019-41439-z)
Supplement: Supplementary file 1 — Supplementary Information [file 41598_2019_41439_MOESM1_ESM.pdf]

The Production of Matchout-Deuterated Cholesterol and the Study of Bilayer-Cholesterol Interactions

Sarah Waldie[1,2], Martine Moulin[1], Lionel Porcar[1], Harald Pichler[3,4], Gernot A. Strohmeier[3,5], Maximilian Skoda[6], V. Trevor Forsyth[1,7], Michael Haertlein[1*], Selma Maric[2,8*], Marité Cárdenas[2*]

1. Institut Laue-Langevin, 71 Avenue des Martyrs, 38042 Grenoble, Cedex 9, France
2. Biofilm-Research Centre for Biointerfaces and Biomedical Science Department, Faculty of Health and Society, Malmo University, Malmo 20506, Sweden
3. Austrian Centre of Industrial Biotechnology, Petersgasse 14, 8010 Graz, Austria
4. Graz University of Technology, Institute of Molecular Biotechnology, NAWI Graz, BioTechMed Graz, Petersgasse 14, 8010 Graz, Austria
5. Graz University of Technology, Institute of Organic Chemistry, NAWI Graz, Stremayrgasse 9, 8010 Graz, Austria
6. Rutherford Appleton Laboratory, Harwell, Didcot OX11 0QX, UK
7. Life Sciences Department, Faculty of Natural Sciences, Keele University, Staffordshire ST5 5BG, UK
8. MAX IV Laboratory, Fotongatan 2, 225 92 Lund, Sweden.

*Corresponding Authors
Michael Haertlein: haertlein@ill.fr
Selma Maric: selma.maric@mau.se
Marité Cárdenas: marite.cardenas@mau.se

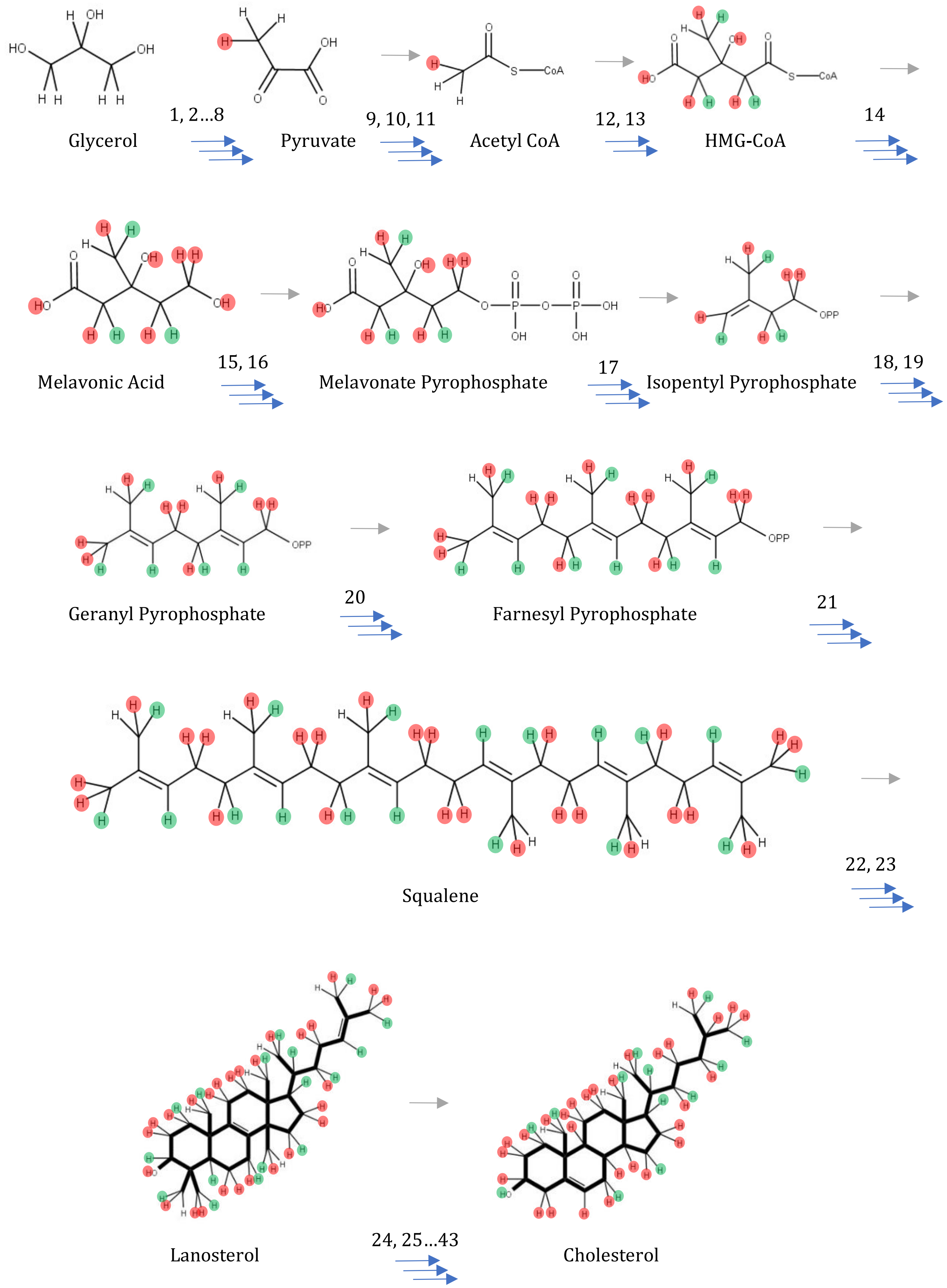

Glycerol
1, 2…8
Pyruvate
9, 10, 11
Acetyl CoA
12, 13
HMG-CoA
14
Melavonic Acid
15, 16
Melavonate Pyrophosphate
17
Isopentyl Pyrophosphate
18, 19
Geranyl Pyrophosphate
20
Farnesyl Pyrophosphate
21
Squalene
22, 23
Lanosterol
24, 25…43
Cholesterol

SI1. Cholesterol biosynthetic pathway. Exchanged protons are given with red or green circles. The red represents deuterium, the green represents the possibility of deuterium or protium. In the synthesis from glycerol to pyruvate there are 8 enzymes involved (1-8): glycerol kinase, glycerol 3-phosphate dehydrogenase, triosephosphate isomerise, glyceraldehyde 3-phosphate dehydrogenase, phosphoglycerate kinase, phosphoglycerate mutase, enolase and pyruvate kinase. From pyruvate to Acetyl Co-enzyme A (CoA) there are three enzymes involved (9-11): pyruvate dehydrogenase, dihydrolipoyl transacetylase and dihydrolipoyl dehydrogenase which all come under the pyruvate dehydrogenase complex. For the conversion of Acetyl CoA to 3-hydroxy-3-methylglutaryl-CoA (HMG-CoA) there are two enzymatic pathways (12-13): thiolase and HMG-CoA synthase. From HMG-CoA to Melavonic acid HMG-CoA reductase is present (14). From melavonic acid to melavonate pyrophosphate there are two enzymes involved (15-16): mevalonate kinase and phosphomevalonate kinase. From melavonate pyrophosphate to isopentyl pyrophosphate (IPP) only one enzyme is used (17): mevalonate-5-pyrophosphate decarboxylase. To convert between IPP and dimethallyl pyrophosphate (DMAPP) the isopentenyl pyrophosphate isomerase enzyme is required (18). To then progress to geranyl pyrophosphate (GPP), farnesyl diphosphate synthase (FPPS) is used (19). To further progress to farnesyl pyrophosphate (FPP) the same FPPS enzyme is required (20). From FPP to squalene the enzyme farnesyl diphosphate farnesyltransferase is used (21). From squalene to lanosterol two further enzymes are required (22-23): squalene monooxygenase and oxidosqualene cyclase. There are a further 19 steps from lanosterol to cholesterol including demethylation, desaturation and reduction among others (24-43).

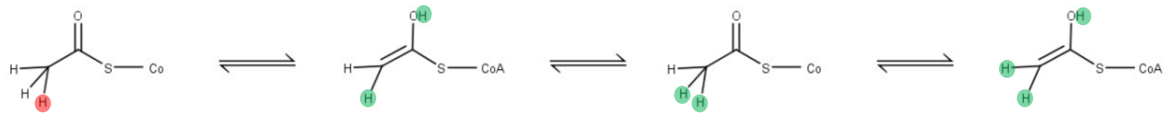

SI2. Tautomerisation of Acetyl CoA.

Table S1. All structural parameters obtained from fitting of derived model to the reflectivity curves for the DLPC + 40mol% cholesterol.

DLPC + 40% cholesterol	Thickness/Å	$\rho \times 10^{-6}$/Å^{-2}	Solvent /%	Roughness/
Si		2.07*		
SiO_2	13.3 ± 0.7**	3.47*	22 ± 3**	5.2 ± 0.5**
Solvent	6.0 ± 0.5**	0*	100*	5.1 ± 0.5**
Inner head	7.0 ± 0.3**	1.89*	26 ± 3**	5.7 ± 0.2**
Inner 1/3 core	10.1 ± 0.3**	4.6 ± 0.2**	17 ± 1**	5.7 ± 0.2**
Middle 1/3 core	10.1 ± 0.3**	2.9 ± 0.2**	17 ± 1**	5.7 ± 0.2**
Outer 1/3 core	10.1 ± 0.3**	4.8 ± 0.1**	17 ± 1**	5.7 ± 0.2**
Outer head	7.0 ± 0.3**	1.89*	26 ± 3**	5.7 ± 0.2**
Backing				5.7 ± 0.2**

* Values kept constant during the fitting process.

**The errors are given in table 3 and are calculated using a Monte-Carlo analysis as embedded in the motofit software[27].

Table S2. All structural parameters obtained from fitting of derived model to the reflectivity curves for the POPC + 40mol% cholesterol.

POPC + 40% mochol	Thickness/Å	$\rho \times 10^{-6}$/Å^{-2}	Solvent /%	Roughness/
Si		2.07*		
SiO_2	7.4 ± 0.3**	3.47*	5 ± 3**	4.3 ± 0.2**
Solvent	4.6 ± 0.3**	0*	100*	4.2 ± 0.2**
Inner head	7.2 ± 0.2**	1.89*	13 ± 3**	4.1 ± 0.1**
Inner 1/3 core	10.5 ± 0.2**	4.06 ± 0.07**	0.5 ± 0.3**	4.1 ± 0.1**
Middle 1/3 core	10.5 ± 0.2**	1.10 ± 0.08**	0.5 ± 0.3**	4.1 ± 0.1**
Outer 1/3 core	10.5 ± 0.2**	4.83 ± 0.07**	0.5 ± 0.3**	4.1 ± 0.1**
Outer head	7.2 ± 0.2**	1.89*	13 ± 3**	4.1 ± 0.1**
Backing				4.1 ± 0.1**

* Values kept constant during the fitting process.

**The errors are given in table 3 and are calculated using a Monte-Carlo analysis as embedded in the motofit software[27].

Table S3. All structural parameters obtained from fitting of derived model to the reflectivity curves for the DMPC + 40mol% cholesterol.

DMPC + 40% mochol	Thickness/Å	$\rho \times 10^{-6}$/Å^{-2}	Solvent /%	Roughness/
Si		2.07*		
SiO_2	10.4 ± 0.6**	3.47*	9 ± 3**	5.7 ± 0.3**
Solvent	5.2 ± 0.1**	0*	100*	5.8 ± 0.2**
Inner head	8.2 ± 0.6**	1.89*	8 ± 2**	5.1 ± 0.5**
Inner 1/3 core	9.9 ± 0.4**	3.2 ± 0.3**	7 ± 1**	5.1 ± 0.5**
Middle 1/3 core	9.9 ± 0.4**	1.0 ± 0.5**	7 ± 1**	5.1 ± 0.5**
Outer 1/3 core	9.9 ± 0.4**	3.9 ± 0.3**	7 ± 1**	5.1 ± 0.5**
Outer head	8.2 ± 0.6**	1.89*	8 ± 2**	5.1 ± 0.5**
Backing				5.1 ± 0.5**

* Values kept constant during the fitting process.

**The errors are given in table 3 and are calculated using a Monte-Carlo analysis as embedded in the motofit software[27].
